# Follicular Fluid from Infertile Women with Mild Endometriosis Impairs *In Vitro* Bovine Embryo Development: Potential Role of Oxidative Stress

**DOI:** 10.1055/s-0040-1718443

**Published:** 2021-01-28

**Authors:** Vanessa Silvestre Innocenti Giorgi, Rui Alberto Ferriani, Paula Andrea Navarro

**Affiliations:** 1 Human Reproduction Division, Department of Gynecology and Obstetrics, Faculdade de Medicina de Ribeirão Preto, Universidade de São Paulo, Ribeirão Preto, SP, Brazil; 2 Conselho Nacional de Desenvolvimento Científico e Tecnológico, Brasília, DF, Brazil

**Keywords:** infertility, endometriosis, oocyte quality, N-acetyl-cysteine, L-carnitine, infertilidade, endometriose, qualidade oocitária, N-acetil-cisteína, L-carnitina

## Abstract

**Objective** To investigate whether follicular fluid (FF) from infertile women with mild endometriosis (ME) alters in vitro bovine embryo development, and whether the antioxidants N-acetyl-cysteine (NAC) and/or L-carnitine (LC) could prevent such damages.

**Methods** Follicular fluid was obtained from infertile women (11 with ME and 11 control). Bovine oocytes were matured in vitro divided in: No-FF, with 1% of FF from control women (CFF) or ME women (MEFF); with 1.5 mM NAC (CFF + NAC, MEFF + NAC), with 0.6 mg/mL LC (CFF + LC, MEFF + LC), or both antioxidants (CFF + NAC + LC, MEFF + NAC + LC). After in vitro fertilization, in vitro embryo culture was performed for 9 days.

**Results** A total of 883 presumptive zygotes were cultured in vitro. No differences were observed in cleavage rate ( *p* = 0.5376) and blastocyst formation rate ( *p* = 0.4249). However, the MEFF group (12.5%) had lower hatching rate than the No-FF (42.1%, *p* = 0.029) and CFF (42.9%, *p* = 0.036) groups. Addition of antioxidants in the group with CFF did not alter hatching rate ( *p* ≥ 0.56), and in groups with MEFF, just NAC increased the hatching rate [(MEFF: 12.5% versus MEFF + NAC: 44.4% ( *p* = 0.02); vs MEFF + LC: 18.8% ( *p* = 0.79); versus MEFF + NAC + LC: 30.8% ( *p* = 0.22)].

**Conclusion** Therefore, FF from infertile women with ME added to medium of in vitro maturation of bovine oocytes impairs hatching rate, and NAC prevented these damages, suggesting involvement of oxidative stress in worst of oocyte and embryo quality of women with ME.

## Resumo

**Objetivo** Investigar se o fluido folicular (FF) de mulheres inférteis com endometriose leve (ME, na sigla em inglês) altera o desenvolvimento in vitro de embriões bovinos, e se os antioxidantes N-acetil-cisteína (NAC) e/ou L-carnitina (LC) poderiam prevenir possíveis danos.

**Métodos** O FF foi obtido de mulheres inférteis (11 com ME e 11 controles). Oócitos bovinos foram maturados in vitro divididos em: sem FF (No-FF), com 1% de FF de mulheres controle (CFF) ou mulheres com ME (MEFF); com 1,5 mM de NAC (CFF + NAC, MEFF + NAC), com 0,6 mg/mL de LC (CFF + LC, MEFF + LC), ou ambos antioxidantes (CFF + NAC + LC, MEFF + NAC + LC). Depois da fertilização in vitro, o cultivo in vitro de embriões foi realizado por 9 dias.

**Resultados** Um total de 883 zigotos presumidos foram cultivados in vitro. Nenhuma diferença foi observada na taxa de clivagem ( *p* = 0,5376) e na taxa de formação de blastocistos ( *p* = 0,4249). Entretanto, o grupo MEFF (12.5%) teve menor taxa de eclosão de blastocistos do que os grupos No-FF (42,1%, *p* = 0,029) e CFF (42,9%, *p* = 0,036). Adição de antioxidantes no grupo com CFF não alterou a taxa de eclosão ( *p* ≥ 0.56), e nos grupos com MEFF, somente a NAC aumentou a taxa de eclosão [(MEFF: 12.5% *versus* MEFF + NAC: 44.4% ( *p* = 0.02); *versus* MEFF + LC: 18.8% ( *p* = 0.79); *versus* MEFF + NAC + LC: 30.8% ( *p* = 0.22)].

**Conclusão** Portanto, o FF de mulheres inférteis com ME adicionado ao meio de maturação in vitro de oócitos bovinos prejudica a taxa de closão embrionária, e a NAC preveniu esses danos, sugerindo o envolvimento do estresse oxidativo na piora da qualidade oocitária e embrionária de mulheres com ME.

## Introduction

Endometriosis is a benign gynecological disease characterized by the presence and growth of endometrial tissue (glands and stroma) outside the uterus. 1 This disease is associated with infertility: ∼ 30% of infertile women present endometriosis 2 and between 30 and 50% of women with endometriosis have difficulties in becoming pregnant. 3

In cases with advanced endometriosis (moderate or severe, stage III/IV), infertility could be due to pelvic anatomical alterations caused by lesions and adherences. 4 On the other hand, minor endometriosis (minimal or mild, stage I/II) is not associated with marked changes in the pelvic anatomy (American Society for Reproductive Medicine [ASRM], 1997) 4 It is unclear whether, after assisted reproduction technologies (ART), endometriosis has a negative impact on clinical pregnancy and live birth rates. 5 6 7 Findings from the most recent meta-analysis showed that women with and without endometriosis have comparable ART outcomes in terms of live births, whereas those with severe endometriosis have inferior outcomes. 6 On the other hand, classical studies assessing natural conception reported lower cumulative pregnancy rate in women with early-stage endometriosis compared with women with infertility of unknown cause. 8 9

In endometriosis women, menstrual reflux and macrophages have been implicated as potential inducers of oxidative stress (OS) 10 11 which, in turn, is involved in impairment of oocyte quality, and in compromising the reproductive capacity of women with early-stage endometriosis. 12 13

Previous studies demonstrated that follicular fluid (FF) from infertile women with mild endometriosis (ME), when added during in vitro maturation (IVM) causes chromosome misalignment and meiotic spindle alterations in bovine 14 15 and murine 16 oocytes. Alterations in oocytes during IVM may affect in vitro embryo development. 17 18 However, no study to date has evaluated the effect of FF from infertile women with ME on embryo development and the impact of antioxidants on this response.

L-carnitine (LC) is a lysine derivative that clears hydrogen peroxide and products of lipid peroxidation. 19 In mitochondria, LC also facilitates the transport of fatty acids during β-oxidation, a major pathway for Adenosine Triphosphate (ATP) production. 20 N-acetyl-cysteine (NAC) is an amino thiol with immunomodulatory, anti-apoptotic, and antioxidant properties. 21 N-acetyl-cysteine is a precursor of intracellular cysteine and reduced glutathione (GSH), which also is and intracellular antioxidant. 22

As human oocytes and embryos are extremely rare, and their use in invasive studies usually prevents their subsequent use in ART, studies using animal models may be useful for the elucidation of the mechanism by which endometriosis leads to infertility. Our hypothesis is that ME leads to OS, and this leads to infertility due to an impairment of oocyte and embryo quality. Therefore, the aim of the present study was to evaluate the impact of adding FF from infertile women with ME and without endometriosis (control), and antioxidants (NAC and/or LC) to the IVM medium of bovine oocytes on in vitro embryo development (cleavage, blastocyst formation and hatching).

## Methods

The present experimental study used an in vitro bovine model. The present study was approved by the Ethics Committee for Animal Experimentation of the Faculdade de Medicina de Ribeirão Preto, Universidade de São Paulo (FMRP-USP, in the Portuguese acronym) (n° 169/2008) and the Research Ethics Committee of the University Hospital, FMRP-USP (n° 12201/2008).

### Patient Selection and Follicular Fluid Collection

Twenty-two FF samples were obtained between February 2009 and February 2011 from infertile women who underwent ovarian stimulation for intracytoplasmic sperm injection (ICSI) at the Sector of Human Reproduction, Department of Gynecology and Obstetrics of the FMRP-USP.

The endometriosis group consisted of patients with infertility associated exclusively with ME, without other gynecological or clinical conditions. An experienced surgeon diagnosed and classified these women by videolaparoscopy using the criteria of the American Society for Reproductive Medicine (1997). 4 The control group consisted of women with tubal or male factor infertility. All control women also underwent videolaparoscopy as part of the protocol for investigation of marital infertility. None of the controls had endometriosis or any other gynecological diseases.

The exclusion criteria were established to reduce confounding factors that could affect OS and/or oocyte quality. Thus, women with any of the following conditions were excluded: age ≥ 38 years old; body mass index (BMI) ≥ 30 kg/m ^2^ ; serum concentration of follicle stimulating hormone (FSH) on the 3 ^rd^ day of the menstrual cycle ≥ 10 mIU/mL; chronic anovulation; presence of hydrosalpinx or chronic diseases such as diabetes mellitus or any other endocrinopathy; cardiovascular disease; dyslipidemia; systemic lupus erythematosus or any other rheumatologic disease; HIV infection or any other active infection; smoking; and use of vitamins, hormonal or nonhormonal medications during the 6 months before inclusion in the study.

Comparison of the means and standard errors of the means (SEMs) indicated that the endometriosis and control groups had similar age (32.72 ± 0.52 versus 30.63 ± 1.36 years old), FSH concentration on the 3 ^rd^ day of the menstrual cycle (5.02 ± 0.90 versus 5.79 ± 0.62 mIU/mL), number of follicles measuring between 14 and 17 mm (10.09 ± 1.43 versus 6.11 ± 1.52 mm), and number of follicles of at least 18 mm after ovarian stimulation (4.89 ± 0.72 versus 3.11 ± 0.76 mm).

### Protocol of Controlled Ovarian Stimulation

Controlled ovarian stimulation (COS) was performed according to our institutional protocol (long protocol). Pituitary blockade was performed by administering an agonist of gonadotropin releasing hormone (GnRH) (Lupron, Abbott, São Paulo, SP, Brazil). Controlled ovarian stimulation was performed by administering recombinant FSH (Gonal-F, Serono, Geneva, Switzerland; Puregon, Organon, Oss, The Netherlands), and ovulation was induced with human chorionic gonadotropin (hCG) (Ovidrel, EMD Serono, Rockland, MA, USA).

Each patient received a daily subcutaneous injection of 0.5 mg leuprolide acetate (Lupron; Abbott) starting 10 days after the first ultrasound exam before COS. Recombinant FSH (Gonal-F; Puregon, 200–225 units/day) was administered during ovarian stimulation, and follicular growth was monitored. Ovulation was triggered with Ovidrel, and oocytes were retrieved between 34 and 36 hours later.

### Collection and Processing of FF Samples

Follicular fluid was collected into individual sterile tubes preheated to 37°C in the absence of culture medium. The sample was only from the first follicle (mean diameter ≥ 15 mm) of the first ovary punctured, with aspiration of the full follicular content. Only FF with no blood contamination upon visual inspection and with a mature oocyte was used. The samples were centrifuged at 300 g for 10 minutes to remove the remaining cells, and the supernatant was stored at - 80°C in two aliquots for future use. Follicular fluid was collected from 22 infertile women, 11 with ME and 11 with male and/or tubal infertility.

We pooled the 11 FF samples of each group for experiments because we had previously tested these samples individually in a study of the role of FF from women with infertility related to ME. 14 The results of that study indicated no intragroup differences and a homogeneous response in all 11 samples from each group. This previous study 14 also tested the effect of 4 different FF concentrations added to the IVM medium (1%, 5%, 10%, and 15%) and indicated no dose-dependent effect. Thus, we used 1% FF concentration.

### Chemicals and Reagents

All chemicals and reagents were purchased from Sigma Chemical Co (St. Louis, MO, USA), unless otherwise stated.

### Preparation of Antioxidant (N-acetyl-cysteine and L-carnitine) Solutions

The solutions of both anti-oxidants were prepared at 100 × (150 mM NAC and 60 mg/mL LC) using water after passage through a filter with 0.22 μm pore. The NAC concentration used to supplement the IVM was 1.5 mM, 23 and the LC concentration was 0.6 mg/mL. 18

### Oocyte Collection

Bovine ovaries were collected immediately after slaughter and transported in physiological saline maintained at between 35 and 38.5°C. In the laboratory, the ovaries were washed with physiological saline supplemented with an antibiotic, and follicles measuring 2 to 8 mm were aspirated by a 21-gauge needle mounted on a 10-mL syringe. Cumulus-oocyte complexes (COCs), with homogeneous cytoplasm and at least 4 layers of cumulus oophorus cells, corresponding to grades 1 and 2 as described previously, 24 were selected under a stereomicroscope and washed in holding medium (TCM-199 medium containing Hanks salts, HEPES buffer, and L-glutamine (Invitrogen, Gibco Laboratories Life Technologies, Thermo Fisher Scientific, Waltham, MA, USA).

### In Vitro Maturation

Selected COCs were cultured in plates containing 4 wells (NUNC, Thermo Fisher Scientific, Waltham, MA, USA) in groups of 20 per well, with 400 µL of culture medium for IVM at 38.5°C, 95% humidity, and 5% CO _2_ 17 25 in a culture system without mineral oil for between 22 and 24 hours. The IVM medium was TCM-199 with Earle salts and bicarbonate (Invitrogen, Gibco Laboratories Life Technologies, Thermo Fisher Scientific, Waltham, MA, USA) supplemented with 0.4 mM sodium pyruvate, 0.5 µg/mL gentamicin, 5 µg/mL FSH, 5 mg/mL LH, 1 µg/mL estradiol, and 10% fetal calf serum (FCS) (Gibco Laboratories Life Technologies, Thermo Fisher Scientific, Waltham, MA, USA)

### In Vitro Fertilization

In vitro matured oocytes were fertilized in vitro with frozen semen from a single bull (CRV Lagoa, SP, Brazil). Before addition, the frozen semen was thawed in 35°C water for 30 seconds. Swim-up was realized as described by Parrish et al. 26 27

Briefly, the COCs were gently pipetted to remove adhering granulosa cells and to break apart aggregations. The disaggregated COCs were transferred into 50 μL microdrops of fertilization medium (114 mM NaCl, 3.1 mM KCl, 25 mM NaHCO3, 47 mg/L NaH2PO4.H2O, 10 mM HEPES, 10 mM sodium lactate 60%, 1.4 mM caffeine, 0.5 mM MgCl2.6H2O, 2 mM CaCl2.2H2O, 0.4 mM sodium pyruvate, 0.5 µg/mL gentamicin, 6 g/L BSA,10 µg/mL heparin, and 40 µL/mL each of penicillamine, hypotaurine, and epinephrine). Sperm (1 × 106/mL) was added and the medium was maintained for between 18 and 22 hours at 38.5°C in a humidified incubator in air with 5% CO2.

### In Vitro Embryo Culture

The presumptive zygotes were denuded from cumulus cells by gentle pipetting and cultured in 50 μL droplets in CR2aa medium supplemented with 1 mg/mL BSA, 40 μg/mL sodium pyruvate, 5 mg/mL hemi-calcium, 20 μL/mL amino acid solution (0.09 μg/mL glutamine, 0.15 μg/mL alanine, and 0.75 μg/mL glycine), 20 μL/mL Eagle Basal Medium (EBM), 10 μL/mL Eagle Minimum Essential Medium (MEM) and 5% FBS, at 38.5°C in a humidified incubator in air with 5% CO2. The culture was maintained for 9 days after fertilization and the medium was renewed every 2 days.

The number of cleaved embryos was recorded on day 3 (72 hours after insemination [HAIs]), blastocyst production was counted on day 7 (168 HAIs), and hatching of blastocysts was checked on day 9 (216 HAIs).

### Experimental Design

**Fig. 1:**
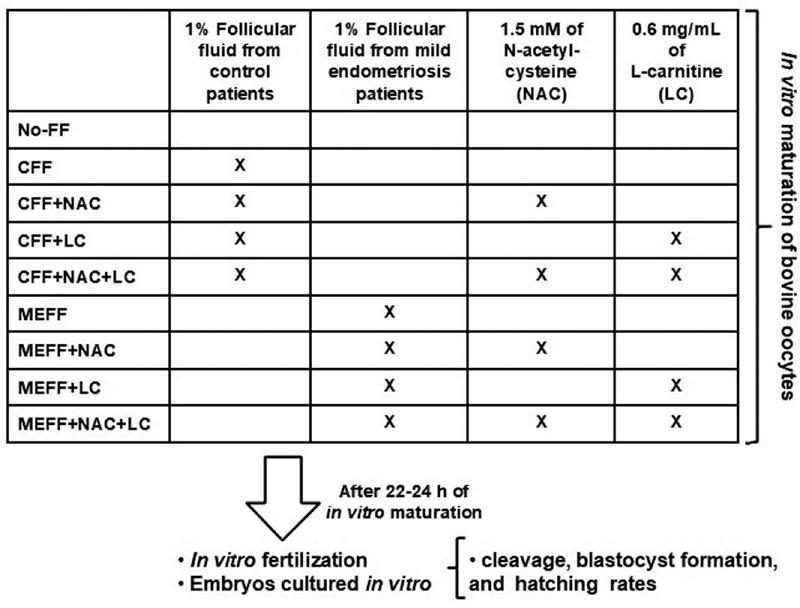
Experimental design.

Immediately after selection, the COCs were subjected to IVM for between 22 and 24 hours, and divided in groups according to the supplementation of IVM medium with FF and antioxidants according to Figure 1 ( Fig. 1 ). After between 22 and 24 hours of IVM, IVF was performed as described above, and embryos were cultured in vitro to assay cleavage, blastocyst formation, and hatching rates.

### Statistical Analysis

Data were analyzed using RStudio software (R Foundation, Vienna, Austria). Categorical variables (cleavage, blastocyst formation and hatching) were expressed as percentage and they were compared by the chi-squared test, considering *p* < 0.05.

## Results

We performed IVF on 898 mature COCs and then cultured 883 presumptive zygotes in vitro. Table 1 shows preimplantation embryo development as cleavage rate, blastocyst formation rate and hatching rate of the different groups.

**Table 1:** Embryo development after *in vitro* fertilization of bovine oocytes which underwent *in vitro* maturation in medium without follicular fluid (No-FF), with 1% FF from infertile control women (CFF), or with 1% FF from infertile women with mild endometriosis (MEFF). The media with CFF and MEFF were supplemented with no antioxidants, 1.5 mM N-acetyl cysteine (NAC), 0.6 mg/mL L-carnitine (LC), or both antioxidants (NAC+LC)

	No − FF	CFF	CFF + NAC	CFF + LC	CFF + NAC + LC	MEFF	MEFF + NAC	MEFF + LC	MEFF + NAC + LC	*p* -value
Presumptive zygotes ( *n* )	100	96	98	98	101	93	97	100	100	
Cleavage rate % ( *n* )	65.00% (65)	61.46% (59)	57.14% (56)	56.12% (55)	60.40% (61)	50.54% (47)	57.73% (56)	56.00% (56)	51.00% (51)	*p* = 0.5376
Blastocysts formation rate % ( *n* )	38.00% (38)	29.17% (28)	26.53% (26)	34.69% (34)	32.67% (33)	25.81% (24)	37.11% (36)	32.00% (32)	26.00% (26)	*p* = 0.4249
Hatching rate % ( *n* )	42.10% (16)	42.86% (12)	50.00% (13)	32.35% (11)	39.40% (13)	12.50% (3) ^a^	44.44% (16)	18.75% (6) ^b^	30.77% (8)	a vs No-FF *p* = 0.029 vs CFF *p* = 0.036 vs MEFF + NAC *p* = 0.020 b vs MEFF + NAC *p* = 0.045

Note: Data were obtained from 5 replicates. Letter indicate *p* value < 0.05 (chi-square test).

a **MEFF** vs No-FF ( *p* = 0.029), *vs* CFF ( *p* = 0.036), *vs* MEFF+NAC ( *p* = 0.02).

b MEFF+LC vs MEFF+NAC ( *p* = 0.045).

No differences were observed between groups in cleavage ( *p* = 0.5376) and blastocysts formation ( *p* = 0.4249) rates ( Table 1 ).

However, in relation to the hatching rate, the groups without FF (No-FF: 42.10%) and with FF from control women (CFF: 42.86%) had a higher hatching rate than the group with FF from ME women (MEFF: 12.50%, versus No-FF: *p* = 0.029; versus CFF: *p* = 0.036).

Addition of antioxidants in groups with FF from control women did not alter the hatching rate [CFF versus CFF + NAC (50.0%, *p* = 0.800), versus CFF + LC (32.35%, *p* = 0.557) and versus CFF + NAC + LC (39.40%, *p* = 0.990)].

Addition of NAC in groups with FF from ME women increased hatching rate (MEFF versus MEFF + NAC: *p* = 0.020), being more efficient than the addition of LC (MEFF + NAC versus MEFF + LC: *p* = 0.045). However, addition of LC and NAC + LC did not alter the hatching rate in groups with FF from ME women (MEFF versus MEFF + LC [18.75%, *p* = 0.793] and versus MEFF + NAC + LC [30.77%, *p* = 0.224]).

Hatching rates in the group with FF from ME women plus the addition of LC or LC + NAC were similar to the groups without FF (No-FF versus MEFF + LC: *p* = 0.066 and versus MEFF + NAC + LC: *p* = 0.511) and with FF from control women (CFF versus MEFF + LC: *p* = 0.080 and versus MEFF + NAC + LC: *p* = 0.524).

## Discussion

The etiopathogenesis of ME-related infertility remains unclear. The present study is the first to show that FF from infertile women with ME impairs bovine embryo development. Besides that, we observed that addition of NAC during IVM can prevent the compromised hatching rate promoted by MEFF.

We observed that FF from ME women added to IVM medium of bovine oocytes did not alter cleavage and blastocyst formation rates, but decreased hatching rate. We expected to find impairment of cleavage in the group with MEFF due to damages on the meiotic spindle of oocytes. 14 15 28 In vivo, cleavage occurs during embryo transit between the oviduct and the uterus, and many factors can potentially interfere with this process. 29 A study using time-lapse showed that meiotic spindle visualization of oocytes is not related to the morphokinetic of in vitro embryo development, but it could be related to clinical pregnancy and live births in women with polycystic ovarian syndrome. 30 A recent study, assessing the impact of endometriosis on embryo development and quality after ICSI, showed similar cleavage rates in groups of women with and without endometriosis, concordant with our findings. 31 In another recent study, Sanchez et al. 31 did not find differences in the blastocyst formation rate comparing control and endometriosis groups undergoing ART. Thus, we hypothesize that mild endometriosis may compromise oocyte quality, 14 15 32 without interfering with cleavage and blastocyst rates, but reducing hatching rate, which could explain lower natural fertility in some of these women. 8 9

Corroborating our findings, Piromlertamorn et al. 33 assessed the impact of incubation of mouse oocytes with endometriotic fluid on early embryo in vitro development, and they also did not show differences in cleavage and blastocyst formation, but they observed that endometriotic fluid impaired the hatching rate. 33 Interestingly, a randomized clinical trial 34 assessing assisted hatching in embryos from women with endometriosis reported higher implantation and clinical pregnancy rates in the group of women whose embryos had undergone laser-assisted hatching after ICSI, suggesting that compromised hatching rate may be involved in lower implantation rates in women with endometriosis. A study, assessing embryo quality and implantation rate in infertile women undergoing ART, showed no differences in the number of good quality embryos between women with and without endometriosis, but reported a statistically significant decrease in implantation rate in the endometriosis group. 35 So, we question whether our findings could explain, at least in part, implantation failure in women with endometriosis and repeated implantation failure after IVF and embryo transfer, which needs further investigation.

Hatching is a prerequisite for embryo implantation in the endometrium and depends on continuous expansion of the blastocele and thinning and rupture of the zona pellucida. 36 Goud et al. 37 reported a correlation between follicular levels of nitrate (an oxidative end product of nitric oxide) and zona pellucida dissolution time (an indirect marker of thickness of the zona pellucida) of oocytes from a group of women with endometriosis. So, we hypothesize that FF from women with ME, due to OS, may cause alterations on the zona pellucida of the oocyte, 38 which compromises the normal hatching processes.

In relation to the addition of antioxidants, NAC and/or LC did not alter cleavage and blastocyst rates in groups with FF from ME women and only NAC prevented reduced blastocyst hatching rates. Thus, we hypothesize that NAC supplementation prevented OS damages in meiotic spindle and on zona pellucida. N-acetyl-cysteine may also have reduced the disulfide bonds in the zona pellucida and induced expansion of the zona pellucida 39 culminating in hatching. However, a study using a rat model demonstrated that intravenous NAC in high concentration (1000 mg.kg-1/day) promotes infertility probably due to an exacerbated thinning of the zona pellucida. 40 Thereby, further studies using animal models are needed to evaluate the effect of different concentrations of NAC to determine its efficacy and safety for embryos before its effects can be evaluated in infertile women with endometriosis.

Interestingly, but corroborating our previous study, 15 concomitant addition of NAC and LC had equal or inferior results relative to NAC or LC alone. We can propose one hypothesis to explain this finding: an interaction of the reactive portions of NAC and LC may have occurred when these antioxidants were solubilized together in aqueous medium, thereby reducing the efficacy of the clearance of free radicals.

Using bovine oocytes and FF from ME women, we aimed to mimetize what could happen in the follicular microenvironment of women with mild endometriosis in natural cycles. However, data obtained from studies using animal models cannot necessarily be extrapolated to humans, and studies evaluating in vitro development of embryos from ME women undergoing ART would be important to confirm our findings. On the other hand, it is important to state that FF obtained from stimulated cycles not necessarily can represent FF from a natural cycle, which needs further investigation.

Therefore, FF from infertile women with ME added to the medium of IVM of bovine oocytes did not interfere with cleavage and blastocyst rates, but impaired hatching rate. N-acetyl-cysteine prevented these damages, suggesting involvement of OS in the worst of oocyte and embryo quality of women with ME. Further studies evaluating the potential clinical application of our findings are needed, especially in terms of improving natural fertility and/or implantation rates in ME women with recurrent implantation failure.
